# Extracellular vesicles during Herpes Simplex Virus type 1 infection: an inquire

**DOI:** 10.1186/s12985-016-0518-2

**Published:** 2016-04-05

**Authors:** Maria Kalamvoki, Thibaut Deschamps

**Affiliations:** Department Microbiology, Molecular Genetics Immunology, University of Kansas Medical Center, 3901 Rainbow Blvd, Building Hixon, room 3009, Kansas City, KS 66160 USA

**Keywords:** Exosomes, Herpes simplex virus, Extracellular vesicles, STING, Tetraspanins, Innate immunity

## Abstract

Extracellular vesicles are defined as a heterogeneous group of vesicles that are released by prokaryotic to higher eukaryotic cells and by plant cells in an evolutionary conserved manner. The significance of these vesicles lies in their capacity to transfer selected cargo composed of proteins, lipids and nucleic acids to both recipient and parent cells and to influence various physiological and pathological functions. Microorganisms such as parasites, fungi and protozoa and even single cell organisms such as bacteria generate extracellular vesicles. In addition, several viruses have evolved strategies to hijack the extracellular vesicles for egress or to alter the surrounding environment. The thesis of this article is that: a) during HSV-1 infection vesicles are delivered from infected to uninfected cells that influence the infection; b) the cargo of these vesicles consists of viral and host transcripts (mRNAs, miRNAs and non-coding RNAs) and proteins including innate immune components, such as STING; and c) the viral vesicles carry the tetraspanins CD9, CD63 and CD81, which are considered as markers of exosomes. Therefore, we assume that the STING-carrying vesicles, produced during HSV-1 infection, are reminiscent to exosomes. The presumed functions of the exosomes released from HSV-1 infected cells include priming the recipient cells and accelerating antiviral responses to control the dissemination of the virus. This may be one strategy used by the virus to prevent the elimination by the host and establish persistent infection. In conclusion, the modification of the cargo of exosomes appears to be part of the strategy that HSV-1 has evolved to establish lifelong persistent infections into the human body to ensure successful dissemination between individuals.

## Background

### Extracellular vesicles

Cells have developed different mechanisms for intercellular communication. Three pathways that have been studied include: a) cell to cell contact; b) secretion of molecules; and c) extracellular vesicles (EVs). This last mechanism was described for the first time in 1983 by two groups [1–3]. Since the discovery of EVs a wealth of information has underscored their impact in normal and pathological processes.

Extracellular vesicles (EVs) is a broad term that describes a heterogeneous group of vesicles released from the cells [4–7]. Based on their origin they are broadly classified into 3 main groups: a) microvesicles/microparticles/ectosomes that are produced from the plasma membrane by outward budding and fission and their size ranges from 100 to 1000 nm; b) apoptotic bodies that are released as blebs from cells undergoing apoptosis and they range from 1000 to 5000 nm; and c) exosomes that are formed by invagination and inward budding of vesicles in the lumen of early endosome, resulting in the formation of multivesicular bodies (MVBs), also called multivesicular endosomes (MVEs) [4, 5]. The MVBs fuse with the plasma membrane releasing the exosomes to the extracellular space. The size of the exosomes ranges from 40 to 100 nm. The major biogenesis pathway of the intraluminal vesicles involves the endosomal sorting complex required for transport (ESCRT) machinery [6–10]. The ESCRT-0 complex and its partner Hrs are involved in the sequestration of ubiquitinated cargo but also in the recruitment of the ESCRT-I complex by interacting with TSG101. ESCRT-I and –II complexes appear to be responsible for the budding and ESCRT-III for the scission of the vesicles in the lumen of the endosomes [11–14]. The MVBs can either fuse with lysosomes ending in the degradation of their content, or follow a secretory pathway leading to the release of the exosomes [15]. The mechanism underlying the fate of MVBs is not yet fully understood, but involves in part the lipid composition of exosomes [4, 9, 15]. MVBs enriched in cholesterol are more likely to follow the exocytic pathway, whereas cholesterol-poor MVBs are targeted to the lysosomes [15]. Ceramide appears to trigger the budding of exosomes into MVBs [16]. Moreover, lysobisphosphatidic acid is enriched in endosomes targeted for degradation but absent from exosomes.

Initially, EVs were viewed as means for cells to dispose of unwanted components. However, in the intervening decade this view has largely changed and it is clear that the cargo of the EVs and the type of recipient cells determine their function [4, 5, 17]. The cargo of the EVs consists of nucleic acids, proteins and lipids. The nucleic acids in EVs are mainly miRNAs, mRNAs and non-coding RNAs [18–24]. These nucleic acids are not randomly incorporated into EVs but rather are preferentially packaged [25, 26]. Moreover, the RNAs in EVs do not reflect the quantity of RNAs in the cells where they originated. Strikingly, the transcripts are intact and they can be translated inside the recipient cells to influence gene expression [25, 26]. For this reason the EVs have emerged as carriers of genetic information that are able to modify the pattern of gene expression in recipient cells. The proteins found in EVs are mainly from endosomes, the plasma membrane and the cytosol. Proteins from organelles such as nucleus, mitochondria, endoplasmic reticulum and the Golgi complex are largely absent. These observations highlight the specificity of the cargo of these vesicles [17]. Lipids, particularly sphingomyelin, cholesterol and hexosylceramides are enriched in EVs at the expense of phosphatidylcholine and phosphatidylethanolamine. Both saturated and monounsaturated fatty acids are also enriched in EVs [6, 16, 27–31].

With respect to the functions of EVs, organisms from different domains of life secrete extracellular vesicles to disseminate information to remote sites from the place of their origin, influencing the surrounding microenvironment through a paracrine mechanism, or altering physiological functions through long distance targeting via the systemic circulation [5]. The composition of EVs is subjected to dramatic changes following alterations in the extracellular environment or different physiological or differentiation stages of the secreting cells. For example, inflammatory signals or activation of innate immunity strongly affect the composition of EVs released by immune cells [32]. In hypoxic conditions of some tumors, the acidic environment or expression of oncogenes radically changes the cargo of the EVs. Moreover, exosomes may contribute to intercellular exchange and spread of prions and misfolded proteins associated with neurodegenerative diseases [33–35]. Based on these properties, EVs are exploited today as diagnostic tools especially in cancer to determine the status of the tumor or its origin, and as vehicles for the delivery of therapeutic small interfering RNAs (siRNAs) [36].

### The extracellular vesicles during viral infections

Many viruses have evolved strategies that rely on the properties and functions of the extracellular vesicles to evade the host [5, 37]. The exosomes released during HIV-1 infection were the first to be studied [37–39]. In 2006, the Trojan exosome hypothesis proposed that retroviruses hijack the exosome biogenesis pathway to release infectious particles encapsulated into exosomal vesicles. Pathways of exosome uptake are utilized for a viral receptor-independent, envelope-independent mode of infection [40]. For example, exosomes derived from dendritic cells, can carry HIV-1 virions that escaped phagolytic and proteasomal degradation. As dendritic cells migrate to the lymph nodes, to present pathogen-derived epitopes to CD4^+^ T or CD8^+^ T lymphocytes, they can transfer HIV-1 to T cells through exosomes, without *de novo* infection [40, 41]. However, the nature of vesicles delivering HIV-1 today is debatable since the virus budding sites coincide with sites of microvesicle biogenesis. Unspliced HIV-1 RNA species but not single- or double-spliced HIV-1 RNAs have been found in exosomes derived either from HIV-1 infected cells or patients [42]. The viral transactivating response element (TAR), which enhances viral replication in recipient cells, is also present in exosomes [43]. Several viral miRNAs including vmiR88, vmiR99 and vmiR-TAR have been detected in exosomes secreted from HIV-1 infected cultures, or isolated from sera of patients [44]. The exosomal vmiR-TAR prevents apoptosis in recipient cells and thereby promotes the infection, whereas the other two miRNAs stimulate release of pro-inflammatory cytokines, such as TNF-α, from recipient macrophages, which may contribute to AIDS pathogenesis [44]. Besides RNA transcripts, many proteins have been detected in EVs derived from HIV-1 infected cells, among them the HIV-1 co-receptors CCR5 and CXCR4, which upon delivery to co-receptor null cells allow HIV-1 entry [45, 46]. This is may be a strategy of HIV-1 to modify its tropism in an otherwise non-permissive background. Additionally, APOBEC3G (A3G), a cytidine deaminase, which is part of the innate host defense system against HIV-1 and other retroviruses, was found in exosomes [47, 48]. Exosomal A3G could confer resistance to both Vif-defective and wild type HIV-1 in exosome recipient cells, suggesting that the restriction was through a non-enzymatic mechanism [49, 50]. Several studies point to the existence of editing-independent activities of A3G that may contribute to its antiviral function [51, 52]. The growing list of proteins found in the exosomes released from HIV-1 infected cells include CD86, CD45 and MHC class II, which may help suppressing immune responses thereby facilitating the virus replication [53].

HIV-1 proteins Gag and Nef have also been shown to be packaged in exosomes [37, 38]. HIV-1 Nef is one of the earliest and most abundantly expressed proteins of HIV-1. Nef was found in exosomes of infected cells in cultures and also in plasma-derived exosomes from patients [38, 54]. Exosomal Nef activates resting CD4^+^ T cells, rendering them permissive to HIV-1 infection, and this way it stimulates the spread of the virus [38, 55]. Nef also increases the exosome production in HIV-1 infected or Nef-transduced cells [56]. In addition, by interacting with vesicular sorting and trafficking pathways it directs MHC-I, CD4 and possible other proteins to MVB for lysosomal degradation, thereby promoting virus replication [57, 58]. Finally, Nef can modulate the miRNA composition of exosomes [59].

Other RNA viruses also hijack the exosomes [37, 39, 60–65]. The exosomes derived from the hepatocytes or from the sera of the hepatitis C virus (HCV) infected patients carry the single-stranded, positive-sense viral RNA genome, and mediate receptor-independent HCV transmission to permissive cells, leading to productive infection [39, 61–65]. The replication competent, negative-stranded viral RNA is also detected in the exosomes [66]. The tetraspanin CD81 is an integral membrane protein and exosome marker, which also serves as a viral entry receptor for HCV. It forms a complex with the viral envelope protein E2 and facilitates its cellular and intercellular trafficking [67, 68]. The HCV genome and the CD81-E2 complex exit cells inside exosomes where they circulate and exploit the fusogenic capabilities of these vesicles to infect naïve cells. Neutralizing antibodies do not interfere with this mechanism of virus spread [67]. Another example is the non-enveloped hepatitis A virus (HAV), whose nucleocapsids were found into vesicles derived from endosomal compartments [60, 69]. This cloaked virus not only was fully infectious but was totally protected from neutralizing antibodies [60, 69]. The virus, via the interaction of the capsid protein VP2 with Alix and the contribution of the VPS4B, two ESCRT-III components, utilizes the exosomes biogenesis machinery to release non-enveloped HAV [60, 69]. Occasionally HAV hijacks membranes and encapsulated virions are released, in an Alix and VPS4B –dependent mechanism [60]. In patients with acute hepatitis A infection, the encapsulated virions were shown to be the dominant form of HAV detected in serum [60, 70]. Antibodies directed against the viral capsid effectively neutralize non-enveloped HAV but did not affect enveloped virus infection [60]. It is possible that the encapsidation of HAV into exosomes is a strategy of the virus to disseminate while escaping immune detection.

Human tumor viruses such as the Epstein-Barr virus (EBV) utilize exosomes to influence the intercellular communication [37, 71]. EBV virus rapidly establishes latent infection in its preferred target cells, the human B lymphocytes, and for this reason the exosomes from these lymphocytes have been most studied. During latency only few viral genes are expressed. The latent membrane protein 1 (LMP1) of EBV is considered the major oncogene and is expressed in multiple human malignancies. LMP1 functions as a constitutive active member of the tumor necrosis factor receptor family, inducing genes that are involved in pro-inflammatory responses, apoptosis, cell proliferation, migration and cell cycle progression [72, 73]. Exosomes released from nasopharyngeal carcinoma (NPC) cells positive for EBV, in which the latency II program of the virus is expressed, contain LMP1 [71], viral miRNAs and signal transduction molecules, such as the epidermal growth factor receptor EGFR [71], galectin-9 [74], fibroblast growth factor (FGF-2) [75], deoxyuridinetriphosphatase (dUTPase) [76]. These exosomes manipulate the tumor microenvironment to enhance tumor progression and alleviate immune responses in tumor cells.

Similar to EBV, the human Kaposi sarcoma virus (KHSV) is associated with multiple lymphomas. Both viruses alter the content of exosomes to modulate cell death and protein synthesis. Analysis of the cargo of exosomes derived from EBV or KHSV-latently infected B lymphocytes demonstrated that approximately one third of the proteins found in the exosomes were unique to the latently infected cells [77]. The functions of these proteins are associated with cancer, cell survival, cell death and disease [77]. Exosomes produced from KSHV-infected primary effusion lymphoma (PEL) cells are highly enriched with enzymes from the glycolytic pathway and at least in B cells they promote glycolysis [77]. These enzymes include pyruvate kinase, enolase, glyceraldehyde dehydrogenase, phosphoglucose isomerase and others. Therefore, a legitimate hypothesis is that exosomal transfer of glycolytic enzymes could enhance glycolysis in recipient cells [78, 79]. In addition, the ribosomal subunits 40S and 60S and several translation initiation factors were found to be increased in KSHV–infected PEL cells, which are most likely through the function of viral proteins K1 and viral G protein that are known to modulate the cellular protein synthesis machinery [77]. Although histones have been shown to be present is exosomes from different cell types, the exosomes from KSHV-infected PEL cells show a preferential increase in histones H1, H2A, H2B, H3 and H4 [77]. The KSHV-infected PEL exosomes also influence adherens junctions of epithelial cells and thus contribute to viral persistence and pathogenesis [77]. Overall, the exosomes produced from KSHV-infected lymphomas appear to exacerbate disease progression and pathogenesis. An intriguing observation was that the nuclear DNA sensor IFI16 is packaged in exosomes and delivered from latently infected KHSV cells to uninfected cells. Activation of IFI16 leads to pro-inflammatory and IFN responses. IFI16 is restriction factor for HSV-1 and 2 and HCMV [80–83]. How exosomal IFI16 could impact immunity to herpes viruses remains elusive.

With respect to the exosomes produced during the lytic cycle of gamma-herpesviruses, recent studies demonstrated that during EBV infection a pre-latent phase precedes the stable latent phase [84]. During the pre-latent phase the virus expresses a subset of immediate-early, early and latent genes, including viral homologues of the anti-apoptotic Bcl-2 family members, the viral interleukin (vIL-10) and BZLF1 that secure the initial success of the EBV infection by blunting immunity and facilitating latency establishment [84, 85]. Additionally, during the pre-latent phase of the infection the EBV particles and the non-viral vesicles that are released from the cells contain viral RNAs of different classes that are delivered to target cells. The packaged viral mRNAs are intact, they are translated in the recipient cells and along with the non-coding RNAs induce viral and cellular genes that potentially modify pathways related to innate and adaptive immune responses [84, 85]. For instance, translation of delivered BZLF1 transcripts could activate resting cells and induce cell-cycle entry, translation of BHRF1 and BALF1 delivered transcripts might protect the infected cells from cell death, delivered miRNAs might control detrimental antiviral responses of the newly infected cells and translation of secreted viral IL-10 mRNAs most likely protect EBV-infected cells from antiviral responses of the innate and adaptive immune system [86–88]. Additionally, the immunoevasins (vIL-10, BGLF5, BNLF2a), expressed into the recipient cells following delivery of their mRNAs within vesicles that are released from the pre-latent EBV infected cells, could protect the newly infected cells from antigen-specific T-cell responses that might otherwise eliminate the newly infected cells before latency can be established [84, 85].

Taken together, extracellular vesicles are released during the productive and the latent stages of gamma-herpesviruses infection but the cargo of these vesicles is substantially different. During the productive cycle the cargo contributes to the success of infection, it primes the cells for persistent infection and prevents the elimination of the virus by the host’s immune system, while the cargo delivered from latently infected cells contributes to virus persistence.

Other herpesviruses modulate the cargo of exosomes. Herpes simplex virus glycoprotein B expressed during the lytic cycle perturbs the endosomal sorting and trafficking of HLA-DR (DR) receptors [89]. Glycoprotein B binds to the DR groove and inhibits the association of peptides to the DR heterodimer [89]. Both proteins co-localize in MVBs and together with CD63 the three proteins are released into the supernatant of infected cells, presumably through the exosomal pathway [89]. The delivery of this complex to recipient cells could modulate immune responses to viral antigens. Human herpes virus 6 (HHV-6) induces formation of MVBs and both viral glycoproteins gB and gM were found in the intraluminal vesicles [90]. Similar to HSV, DR and CD63 along with the glycoproteins gB of HHV-6 are packaged in exosomes and delivered to target cells [89].

The emerging roles of different types of extracellular vesicles and particularly of exosomes in infectious diseases could provide information about pathogens and their strategies for dissemination.

### The extracellular vesicles in herpes simplex virus-infected cells

During herpes simplex virus infection different kind of vesicles appear to be released extracellularly. Szilagyi and Cunningham reported that in addition to the virions, also known as H (Heavy)-particles, other particles named L (Light)-particles are released [91]. Microvesicles, is an alternative term utilized frequently for the L-particles although with the current knowledge on EVs the term might not be accurate. The L-particles are composed of virus envelope and tegument proteins but they lack viral genome and viral capsid proteins. The L-particles cover a wide range of sizes and often contain inclusion vesicles of variable size and number [91–93]. Although the L-particles are non-infectious they were shown to facilitate the HSV-1 infection, at least in cell cultures, most likely by delivering viral proteins such as ICP0 and ICP4 to the target cells and possibly cellular factors that are needed for virus replication and suppression of antiviral responses [91–94].

Apoptotic bodies have been reported on certain occasions during herpes simplex virus infection although several HSV genes are known to block apoptosis. Thus, neonatal neutrophils on infection break up into multiple apoptotic bodies that contain live virus and they may facilitate the spread of HSV as the apoptotic bodies are engulfed by macrophages [95]. Apoptotic bodies might also be released by neuronal cells undergoing apoptosis during HSV infection [96–98]. The size of apoptotic bodies as reported earlier ranges between 1000 and 5000 nm.

From this point forward the focus of the review will be on extracellular vesicles with a size range between 50 and 110 nm that are released from the HSV infected cells and they have properties similar to exosomes, that is, they carry the exosomal markers CD63, CD9 and CD81 and are smaller than apoptotic bodies or microvesicles. The concept that extracellular vesicles are released upon infection having properties similar to exosomes is based on the observations discussed below:

STING (**ST**imulator of **In**terferon **G**enes) is a sensor of DNA in the cytoplasm, which has functions hostile to the virus in normal cells and in mice that impede virus replication and dissemination [99–101]. However, in a number of cancer derived cell lines such as human cervical carcinoma (HeLa) and human epithelial (HEp-2) STING was protected from elimination by the wild type HSV-1. This conclusion emanated from the observation that STING was rapidly eliminated from these cells following infection by HSV mutants impaired in the execution of late viral functions such as the ICP0 E3 ligase activity and the ICP0- null mutant, a Us3 kinase-deficient mutant and the ΔICP4 replication-deficient mutant [102]. These data suggested that the functions of ICP0 and Us3 were required to protect STING from elimination [102]. Moreover, experiments that assessed the growth of the wild type HSV-1 and the ICP0 null- mutant in normal immortalized (human embryonic lung fibroblasts; HEL) and cancer cells (epithelial HEp-2) depleted of STING, demonstrated that although STING was detrimental to both viruses in the normal cells, it was required for optimum replication for both viruses in the cancer cells [102]. Taken together, these data suggested that STING, under certain conditions, might be utilized by HSV-1 [102].

A clue as to what additional functions might STING perform during HSV-1 infection emerged from the observation that in Vero cells (African green monkey kidney epithelial cells) the endogenous level of STING was very low. Following exposure to different doses of the wild type virus, STING was detectable in infected cells as soon as 30 minutes post-inoculation and reached a plateau at two hours post-exposure where it remained stable up to18 h post-inoculation [103]. The accumulation of STING in Vero cells was proportional to the dose of the virus and was not related to changes in the abundance of STING transcripts, as it remained stable through the course of the infection [103]. Further, inhibition of protein synthesis did not alter the accumulation of STING in HSV-1-infected Vero cells [103]. These data suggested that accumulation of STING in Vero cells was due to the virus inoculum and not due to stimulation of its gene expression.

Indeed, HSV-1 virions purified through a dextran-10 gradient, as described before, were found to contain both the monomeric and a dimeric forms of STING [103]. This observation raised two possibilities, either that STING was incorporated in HSV-1 virions or that it was present in separate structures co-purifying with the virions. Several experiments were designed to address this issue. First, immunoprecipitation reactions with the STING antibody were carried out using dextran-10 gradient purified virions. This approach yielded negative results as STING remained in the supernatant of the reaction along with virion components. Two possible scenarios could explain these results, either STING was indeed incorporated into HSV-1 virions, or the STING epitope, in the structures where STING was integrated, was not accessible to the antibody and as a consequence the protein remained in the supernatant. To distinguish between these two possibilities a similar immunoprecipitation reaction was carried out using an antibody against the tetraspanin CD9. CD9 is a common marker of the exosomes and it forms heterooligomers with other members of the tetraspanin family, such as CD63, another exosomal marker. The results of this reaction indicated that STING was in structures separate than the virions, as the majority of STING co-immunoprecipitated with CD9, while the virion components remained in the supernatant [103]. To verify the above results, the presumed virions/exosomes mixture was incubated with antibody against the viral glycoprotein gD, to neutralize the virus, and subsequently the mixture was added to Vero cells, whose endogenous STING is negligible. The rationale was that the gD antibody would block viral entry, while the fate of STING was expected to be independent of gD. Indeed, the neutralized virus could not enter the cells and viral gene expression was not detected. However STING was delivered in Vero cells, in the presence of the neutralizing gD antibody, even when protein synthesis was blocked by the addition of cycloheximide [103]. These data supported the observation that STING was not incorporated in HSV-1 virions and further demonstrated that STING entered the cells via a mechanism independent of the viral entry. As an alternative approach, the release of STING and CD9 was monitored in the supernatant of cultures infected with a HSV-1 ΔUL18 mutant that is defective in assembly. UL18 is essential for capsid assembly, and in its absence virion formation does not occur [104]. UL18 is not required for viral gene expression or the virus replication. The assumption was that if STING was in non-virion structures, its release in the supernatant should not be affected. Indeed, this experiment demonstrated that both STING and CD9 were in the culture supernatant of the ΔUL18 mutant infected cells despite the absence of virions. Although there might be differences in the number and molecular composition of the STING-carrying vesicles in the supernatant, cells inoculated with this mutant provided useful information with respect to the presence of STING in exosomes.

It worth mentioning that the Vero cell line which expresses low levels of STING, was identified as a useful system to study delivery of the EVs carrying STING. Interestingly, the level of CD9 (an marker for exosomes) in Vero cells is remarkably low compared to other cell lines, which may be indicative to the number and/or type of vesicles released from these cells.

The previous data not only supported that STING was released in higher-ordered structures in the supernatant of the infected cultures but provided some clues about structural characteristics of these structures. These included that: a) the vesicles could be delivered to target cells, as STING from the virus inoculum was delivered into the recipient cells exposed to the virus; b) the optimal time for delivery was approximately two hours as the levels of STING in the recipient cells gradually increased for the first two hours following exposure; c) the HSV-1 glycoprotein gD was not required for the STING-carrying structures to enter the target cells; d) protein synthesis was not required for their entry; and e) in dextran-10 density gradients, HSV-1 virions and the structures carrying STING co-purified. As it will be discussed later, co-fractionation in some density gradients is most likely due to co-aggregation during high speed sedimentation.

STING has four transmembrane regions and a carboxy-terminal domain, and has been classified as an endoplasmic reticulum (ER) protein, which may associates with mitochondria-associated ER membranes (MAM) at the interface between the mitochondrion and the ER [105–107]. Following activation, STING appears to re-localize from ER to perinuclear vesicles [105–107]. On infection of a HEp-2 cell line stably expressing human STING, the protein was found in globular structures in the perinuclear region and at the poles of the cells**.** Tetraspanins CD63 and CD81 perfectly co-localized with STING in the globular structures [*Kalamvoki et al, unpublished data*]. CD63, the first characterized tetraspanin, is mainly associated with membranes of intracellular vesicles and is abundantly present in late endosomes and lysosomes [4, 108, 109]. CD63 is enriched in the intraluminal vesicles of multivesicular bodies (MVBs), which are secreted as exosomes through fusion of the MVBs with the plasma membrane [109]. Localization of CD63 at the plasma membrane has been described in clusters called tetraspanin-enriched microdomains [108–110]. Similarly, CD81 is another marker of exosomes and an integral component of the plasma membrane found in focal adhesions and occasionally immunological synapses [4, 108].

Taken together, these data suggest that STING is packaged in extracellular vesicles during HSV-1 infection reminiscent to exosomes. These vesicles will be referred as “HSV-1 exosomes or viral exosomes” as their cargo consists not only of host but also viral factors. Below we will describe the most effective approach to efficiently separate the viral exosomes from HSV virions.

### The challenge of separating the HSV-1 exosomes from herpes simplex virus 1 particles

The observation was made that HSV stocks were either enriched or depleted of the STING-carrying vesicles depending on the cell line in which the viral stock was produced [103]. Viral stocks prepared in HEp-2 cells were enriched in STING/CD9-containing vesicles while stocks prepared in Vero cells were largely devoid of STING/CD9-containing vesicles [103]. Thus, the lack of purity and the heterogeneity of the virus inoculum is dependent on the cell line used to propagate the viral stock and should be taken into consideration as they could account for differences in host responses [103].

Several different approaches have been used in an attempt to separate the HSV-1 virions from the STING-carrying vesicles. We will discuss the results of each approach below.

The first approach was based on immunoaffinity. It involved differential centrifugation of the culture supernatants to clarify cells debris and nuclei, followed by sedimentation of virions at high speeds. After washing, to remove protein impurities and small aggregates, the pellet was subjected to immunoaffinity purification using an antibody against the tetraspanin CD9 to precipitate the STING-carrying vesicles, or after removing the HSV-1 virions with antibody against the glycoprotein D (gD). Although this approach clearly demonstrated that STING was in different structures from virions, co-aggregation of virions with vesicles during ultracentrifugation did not yield viral exosomes of the desired purity.

The second approach was based on dextran-10 density gradients [111]. We sought to determine whether a linear dextran-10 gradient (density 1.04-1.09 g/cm^3^) that has been extensively used for partial purification of HSV virions could be utilized to separate the virions from the STING-carrying vesicles, as their densities were speculated to be different. The results of this approach demonstrated that the two structures were inseparable, as they were found in the same fractions of this gradient. We believe that co-aggregation occurring during high speed sedimentation interfered with efficient separation of vesicles from the virions.

A third approach was the utilization of egress-deficient HSV mutants. This approach indeed yielded STING-carrying vesicles free of viral particles. However, whether the composition of their cargo is the same as in wild type virus infected cells, as largely reflected by the environment of its origin, remains a subject of investigation.

**Fig. 1:**
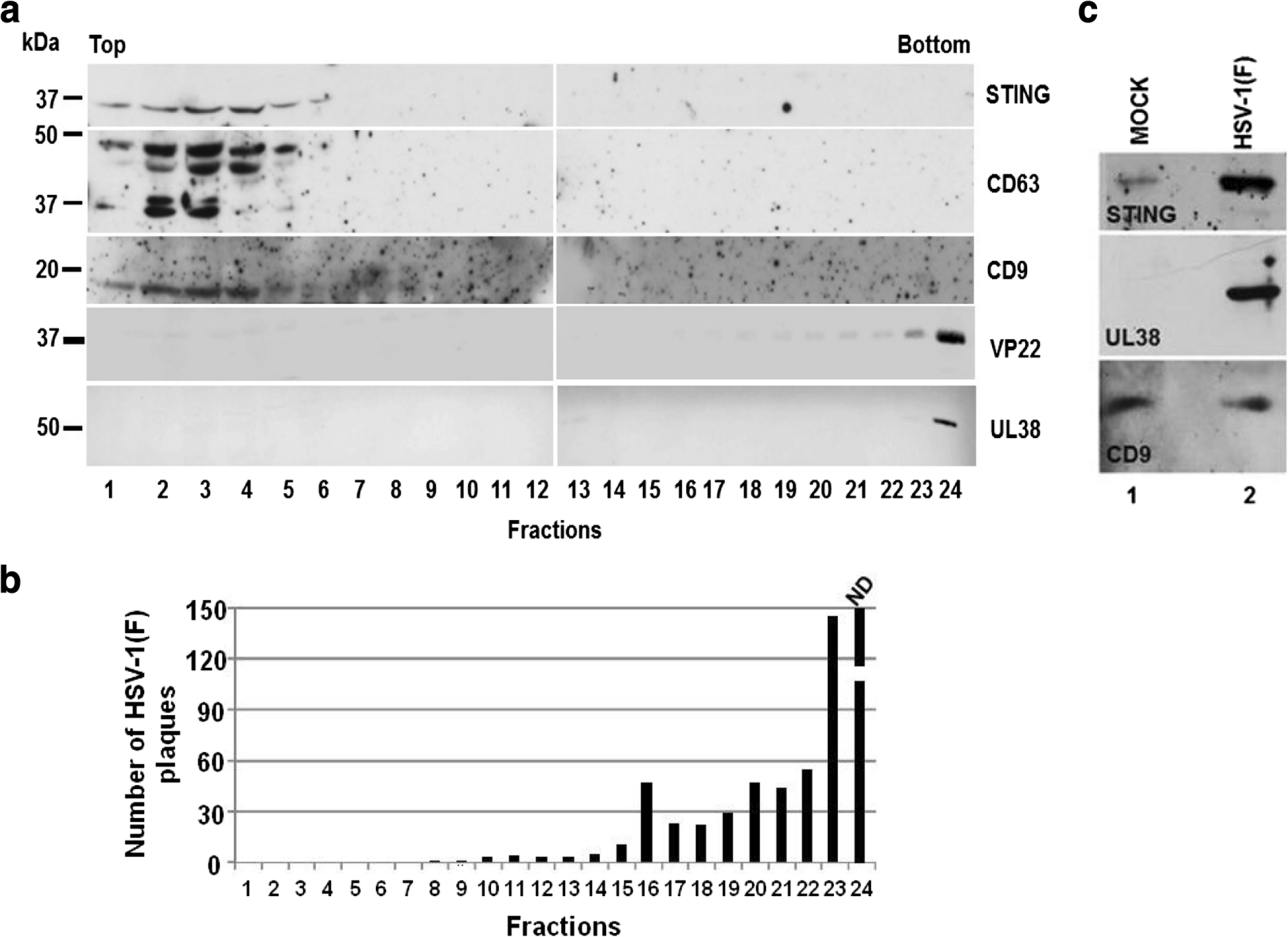
Separation of STING-carrying vesicles from HSV-1 virions. (**a**) Supernatant from human epithelial cells (HEp-2) infected with HSV-1(F) was clarified by differential centrifugation to remove cell debris and nuclei, filtered concentrated before loaded onto a iodixanol gradient, as detailed in *Deschamps T. and Kalamvoki M*, manuscript in preparation. Fractions were collected from the top to the bottom of the gradient and the proteins were identified by immunoblot analysis. The tegument virion protein 22 (VP22) and the capsid unique long 38 protein (UL38) were found in high density iodixanol fractions. STING, CD63 and CD9 were floating in the low density fractions. (**b**) The same fractions were tested for the presence of infectious viral particles, by plaque assay in Vero cells. The number of viral plaques in each fraction were counted after Giemsa staining. (**c**) EVs and virions derived from the supernatant of HEp-2 cells exposed to HSV-1(F) were pelleted before loaded onto a dextran-10 gradient (1.04-1.09 g/cm^3^). The HSV-1 virions and the tetraspanin CD9 along with STING were found in the same fraction

Having identified the limitations of commonly used systems we developed an iodixanol gradient for separation of vesicles from virions (Deschamps T, Kalamvoki M: Characterization of exosomes released from HSV-1 infected cells, in preparation) [112, 113]. The samples were obtained from the supernatant of infected cultures following differential centrifugation at low speeds to sediment cell debris and nuclei, filtration to remove large aggregates followed by filter concentration. This approach resulted in segregation of any kind of HSV particles from the STING-carrying vesicles, as was assayed by immunoblot analysis. The HSV-1 capsid protein unique long 38 (UL38) and the tegument protein 22 (VP22), were found in high density fractions, while STING and the tetraspanins CD9 and CD63 were detected in the low density fractions (Fig. 1, panel a). Consistent with the fractionation results, a plaque assay demonstrated that the infectious viral particles were present only in the high density fractions (Fig. 1, panel b) (Deschamps T, Kalamvoki M: Characterization of exosomes released from HSV-1 infected cells, in preparation). An alternative approach based on continuous dextran-10 gradient failed to segregate the STING-carrying vesicles from the viral particles (Fig. 1, panel c) [111]. In conclusion, best practices to separate the HSV-1 exosomes from HSV-1 virions involve the concentration of cell culture supernatant by avoiding high speed sedimentation, which results in aggregation.

### Potential functions of exosome-like vesicles released from herpes simplex virus infected cells

A clue to the potential functions of the virally-induced exosomes could emerge from the analysis of their cargo. Beside STING, the tetraspanins CD63 and CD81 co-immunoprecipitated with a CD9 antibody from the supernatant of infected cultures, suggesting that STING was incorporated in the tetraspanin-enriched vesicles reminiscent to the exosomes.

In extracellular vesicles, such as exosomes, the cargo is presumably determined by the type of signals the donor cells communicate to the target cells. For this reason it is not surprising that the cargo of these vesicles can be significantly different from the parental cell content. Additionally, despite their limited capacity it has become clear that the miRNAs and intact transcripts contained within these vesicles can potentially influence gene expression in target cells. Similarly, the proteinaceous cargo is sufficient to modulate host responses. For example, in tumors the exosomes released by the cancer cells facilitate tumor growth and metastasis [4, 9, 10, 114–117]. Within infected cells, pathogens modify the cargo of exosomes to create a microenvironment that facilitates their replication, spread and their persistence in the host [5, 37, 39, 64, 71, 118, 119].

Recently, we found that the STING-carrying vesicles released from HSV-infected cells deliver selected viral transcripts, some of which have functions during the latent stage of the virus [103]. Among those identified were the latency associated transcript (LAT), the most abundant non-coding RNA present in latently infected ganglia, and viral microRNAs miR-H5, miR-H3, miR-H6 whose abundance increases during latency but rapidly declines following herpes reactivation [103]. An attractive hypothesis is that the virus releases these transcripts to curtail its dissemination within the host. By preventing the elimination of the host the virus increases its chances to spread between individuals. Consistent with this hypothesis is the fact that components of the innate immunity, such as the DNA sensor STING, are also packaged in the HSV exosomes. One possibility is that the viral exosomes would prime neighboring uninfected cells for antiviral responses, which would subsequently control the spread of the virus.

Transcripts for immediate early viral genes, such as the infected cell protein 27 (ICP27), and late genes, such as the virion protein 16 (VP16), were also detected in the viral exosomes [103]. These transcripts, provided that are expressed, are expected to exert effects on transcription. Although components of the viral exosomes might have opposing functions it is unclear whether the entire population of viral exosomes is homogenous and what functions dominate under certain conditions.

Numerous studies have argued that several host components which co-purify with HSV, HCMV, HIV-1 particles are packaged in the virions. Although such a possibility cannot be excluded for a handful of molecules, as the list of these components increases this scenario becomes less likely. Mass spectrometric analysis of purified HSV-1 virions have identified almost 50 host proteins [120–122]. Notably, many of these are components of the vesicle biogenesis and trafficking pathways. Other studies have argued that numerous host transcripts and several viral transcripts co-purify with HSV-1 and HCMV virions [123–125]. In light of our recent studies, which demonstrated that virions and extracellular vesicles co-purify, the question arises as to whether some of the presumed virion components may actually belong to extracellular vesicles that co-purify with virus. Taken together, it becomes critical to identify the viral and host macromolecules that are packaged inside the “HSV exosomes” and delivered to uninfected cells. This information is important to understand the viral dissemination strategies, identification of the mechanisms of viral latency and provide insight into virus pathogenesis.

## Conclusions

We have discussed a strategy that HSV-1 has evolved to evade the host, which involves alterations in the content of the extracellular vesicles to include components of innate defense against DNA viruses such as STING and selected viral gene products, such as transcripts expressed during the latent stage of the virus. The reorganization of the extracellular vesicles is part of the mission of the virus to alter the environment in the recipient cells to control its dissemination in the host. By restricting its dissemination within the human body, the virus ensures long-term interactions with the host and increased chance of transmission in the population.

Cells generally secrete different types of vesicles. Our focus has been on the STING-carrying vesicles produced during HSV-1 infection. The components of these vesicles also include three tetraspanins, CD9, CD63 and CD81, which are usually present on the membrane of exosomes [4, 108, 109]. For this reason and because of their size range (50 - 110 nm) we refer to them as “HSV-1 exosomes”. A few viral transcripts were found in these vesicles using a targeted approach, but a more systematic approach is in progress to identify the nucleic acids and the proteins that constitute their cargo. In the future it will be important to elucidate the roles of individual factors packaged in exosomes during HSV infection. Another issue is how the cargo composition is determined during HSV infection. A small animal model to address the influence of exosomes and of individual exosomal components on HSV pathogenesis will be invaluable.

Several pathogens have evolved mechanisms to hijack and utilize the extracellular vesicles. Some viruses utilize components of the exosome biogenesis machinery for egress while others bud inside extracellular vesicles and traffic to remote sites escaping immune surveillance. There are no evidence so far that herpes virions are packaged inside exosomes. In many instances the cargo of extracellular vesicles is modified to alter the microenvironment of the infection [5, 32, 119].

Extracellular vesicles, including exosomes, have garnered increased attention during the last decade as they constitute a major mechanism for intercellular communication and in pathogenesis of cancer, microbial and viral infections, autoimmune, neurodegenerative diseases and other disease states they appear to exacerbate the outcome of the disease. Many types of these vesicles, including the exosomes, are stable in biological fluids, can be transported to sites remote to the vesicular origin, and they are characterized by unique molecular signatures representing the physiological state of the cells from which they originated [115, 126]. For these reasons, their diagnostic value along with their potency of carrying biomarkers during disease states are under intense investigation [115, 127, 128]. These features, along with the evolution in technologies for segregating, purifying and characterizing the extracellular vesicles, have intensified the research to understand their impact in cell physiology and functions.

## Abbreviations

APOBEC3G (A3G): apolipoprotein B mRNA editing enzyme, catalytic polypeptide-like 3G
BALF1: BamHI A leftward fragment 1
BGLF5: BamHI G leftward frame 5
BHRF1: BamHI fragment H rightward open reading frame 1
BNLF2a: BamHI**-**N leftward frame 2a
BZLF1: BamHI Z fragment leftward open reading frame 1
CCR5: C-C chemokine receptor type 5
CD4: cluster of differentiation 4
CD45: cluster of differentiation 45
CD63: cluster of differentiation 63
CD81: cluster of differentiation 81
CD86: cluster of differentiation 86
CD9: cluster of differentiation 9
CXCR4: C-X-C chemokine receptor type 4
EBV: epstein- barr virus
EGFR: epidermal growth factor receptor
ER: endoplasmic reticulum
ESCRT: endosomal sorting complex required for transport
EVs: extravesicular bodies
FGF-2: fibroblast growth factor-2
gD: glycoprotein D
gM: glycoprotein M
HAV: hepatitis A virus
HCMV: human cytomegalovirus
HCV: hepatitis C virus
HEV: hepatitis E virus
HHV-6: human herpesvirus 6
HIV-1: human immunodeficiency virus type-1
HLA-DR: human Leukocyte Antigen - antigen D Related
Hrs: hepatocyte growth factor regulated tyrosine kinase substrate
HSV-1: herpes simplex virus type 1
ICP0: infected cell protein 0
ICP27: infected cell protein 27
ICP4: infected cell protein 4
IFI16: gamma-interferon-inducible protein 16
LAT: latency associated transcript
LMP1: latent membrane protein 1
MAM: mitochondria-associated membrane
MHC I or II: major histocompatibility complex class I or II
miRNA: micro ribonucleic acid
mRNA: messenger ribonucleic acid
MVBs: multivesicular bodies
MVEs: multivesicular endosomes
NPC: nasopharyngeal carcinoma
PEL: primary effusion lymphoma
RNA: ribonucleic acid
siRNA: small interfering RNA
STING: stimulator of interferon genes
TAR: transactivating response element
TSG101: tumor susceptibility gene 101 protein
UL18: unique long region 18 protein
UL38: capsid protein unique long 38
Us3: unique short region 3 protein.
Vif: viral infectivity factor
vIL-10: viral interleukin 10
VP16: virion protein 16
VP22: virion protein 22
VPS4B: vacuolar protein sorting 4 homolog B (S. cerevisiae)
